# What Are the Sex-Based Differences of Acetabular Coverage Features in Hip Dysplasia?

**DOI:** 10.1097/CORR.0000000000003126

**Published:** 2024-07-12

**Authors:** Hiroto Funahashi, Yusuke Osawa, Yasuhiko Takegami, Hiroki Iida, Yuto Ozawa, Hiroaki Ido, Shiro Imagama

**Affiliations:** 1 Department of Orthopaedic Surgery, Nagoya University Graduates School of Medicine, Nagoya, Japan

## Abstract

**Background:**

Eccentric rotational acetabular osteotomy is performed to prevent osteoarthritis caused by developmental dysplasia of the hip (DDH). To achieve sufficient acetabular coverage, understanding the characteristics of acetabular coverage in DDH is necessary. However, the features of acetabular coverage in males with DDH remain unclear. We thought that the differences in acetabular coverage between females and males might be associated with the differences in pelvic morphology between the sexes.

**Questions/purposes:**

(1) What are the differences in the acetabular coverage between females and males with DDH? (2) What are the differences in the rotations of the ilium and ischium between females and males with DDH? (3) What is the relationship between the rotation of the ilium and ischium and the acetabular coverage at each height in females and males with DDH?

**Methods:**

Between 2016 and 2023, 114 patients (138 hips) underwent eccentric rotational acetabular osteotomy at our hospital. We excluded patients with Tönnis Grade 2 or higher, a lateral center-edge angle of 25º or more, and deformities of the pelvis or femur, resulting in 100 patients (122 hips) being included. For female patients (98 hips), the median (range) age was 40 years (10 to 58), and for the male patients (24 hips), it was 31 years (14 to 53). We used all patients’ preoperative AP radiographs and CT data. The crossover sign, posterior wall sign, and pelvic width index were evaluated in AP radiographs. The rotation of the innominate bone in the axial plane was evaluated at two different heights, specifically at the slice passing through the anterior superior iliac spine and the slice through the pubic symphysis and ischial spine in CT data. Furthermore, we evaluated the anterior and posterior acetabular sector angles. Comparisons of variables related to innominate bone measurements and acetabular coverage measurements between females and males in each patient were performed. The correlations between pelvic morphology measurements and acetabular coverage were evaluated separately for females and males, and the results were subsequently compared to identify any sex-specific differences. For continuous variables, we used the Student t-test; for binary variables, we used the Fisher exact test. A p value less than 0.05 was considered statistically significant.

**Results:**

In the evaluation of AP radiographs, an indicator of acetabular retroversion—the crossover sign—showed no differences between the sexes, whereas the posterior wall sign (females 46% [45 of 98] hips versus males 75% [18 of 24] hips, OR 3.50 [95% confidence interval (CI) 1.20 to 11.71]; p = 0.01) and pelvic width index less than 56% (females 1% [1 of 98] versus males 17% [4 of 24], OR 18.71 [95% CI 1.74 to 958.90]; p = 0.005) occurred more frequently in males than in females. There were no differences in the iliac rotation parameters, but the ischium showed more external rotation in males (females 30° ± 2° versus males 24° ± 1°; p < 0.001). Regarding acetabular coverage, no differences between females and males were observed in the anterior acetabular sector angles. In contrast, males showed smaller values than females for the posterior acetabular sector angles (85° ± 9° versus 91° ± 7°; p = 0.002). In females, a correlation was observed between iliac rotation and acetabular sector angles (anterior acetabular sector angles: r = -0.35 [95% CI -0.05 to 0.16]; p < 0.001, posterior acetabular sector angles: r = 0.42 [95% CI 0.24 to 0.57]; p < 0.001). Similarly, ischial rotation showed a correlation with both acetabular sector angles (anterior acetabular sector angles: r = -0.34 [95% CI -0.51 to -0.15]; p < 0.001 and posterior acetabular sector angles: r = 0.45 [95% CI 0.27 to 0.59]; p < 0.001). Thus, in females, we observed that external iliac rotation and ischial internal rotation correlated with increased anterior acetabular coverage and reduced posterior coverage. In contrast, although acetabular coverage in males showed a correlation with iliac rotation (anterior acetabular sector angles: r = -0.55 [95% CI -0.78 to -0.18]; p = 0.006 and posterior acetabular sector angles: r = 0.74 [95% CI 0.48 to 0.88]; p < 0.001), no correlation was observed with ischial rotation.

**Conclusion:**

In males, acetabular retroversion occurs more commonly than in females and is attributed to their reduced posterior acetabular coverage. In females, an increase in the posterior acetabular coverage was correlated with the external rotation angle of the ischium, whereas in males, no correlation was found between ischial rotation and posterior acetabular coverage. In treating males with DDH via eccentric rotational acetabular osteotomy, it is essential to adjust bone fragments to prevent inadequate posterior acetabular coverage. Future studies might need to investigate the differences in acetabular coverage between males and females in various limb positions and consider the direction of bone fragment rotation.

**Clinical Relevance:**

Our findings suggest that males with DDH exhibit acetabular retroversion more frequently than females, which is attributed to the reduced posterior acetabular coverage observed in males. The smaller posterior acetabular coverage in males might be related to differences in ischial morphology between sexes. During eccentric rotational acetabular osteotomy for males with DDH, adequately rotating acetabular bone fragments might be beneficial to compensate for deficient posterior acetabular coverage.

## Introduction

Developmental dysplasia of the hip (DDH) affects between 0.6% and 7.6% of individuals, varying by race, and is associated with an early onset of hip arthritis [21]. These abnormalities cause increased cartilage contact pressure and acetabular labral tears at the joint level, leading to cartilage breakdown [6, 16, 21, 28]. Many procedures have been proposed to address these abnormalities, including hip arthroscopy, femoral osteotomy, and periacetabular osteotomy [17, 19, 29]. Eccentric rotational acetabular osteotomy is a surgical technique that requires osteotomy around the acetabulum and appropriate realignment for symptomatic acetabular dysplasia [10]. A thorough understanding of the individual pelvic morphology [8, 9, 25] and acetabular coverage [22, 33] is crucial for the success of eccentric rotational acetabular osteotomy, and careful attention to the relationship between pelvic morphology and acetabular coverage is needed to achieve the correct amount of acetabular coverage of the acetabular fragment [4, 5]. Therefore, many studies have evaluated the pelvic morphology and acetabular coverage in DDH [7, 16, 22, 24, 33]. However, many of these studies were limited to females, and reports focusing on the differences in pelvic morphology and acetabular coverage between female and male patients with DDH are lacking.

Most studies on dysplasia and pelvic morphology have focused on females [13, 27]. The association between acetabular coverage and the rotation of the innominate bone in females with DDH has been reported [4]. There are considerable individual differences in the rotation of the innominate bone. Understanding these characteristics allows surgeons to anticipate acetabular coverage, providing essential guidance for determining the direction of bone fragment rotation during osteotomies. However, many aspects of pelvic morphology and acetabular coverage in males with DDH remain unclear. The acetabular version in males with asymptomatic hips has been reported to be smaller than in females with asymptomatic hips, and the anterior acetabular coverage in males with asymptomatic hips appears to be larger [27]. Additionally, a few studies have suggested that acetabular anteversion in males with DDH tends to be smaller than in females with DDH [23, 31]. However, no differences in anterior acetabular coverage have been reported between females and males with DDH [5, 24]. If the anterior coverage in males with DDH is larger than in females with DDH, rotating the acetabular fragment forward during eccentric rotational acetabular osteotomy may be a risk factor for anterior impingement during hip flexion [9, 25]. Furthermore, the correlation between acetabular coverage and the rotation of the ilium and ischium in males with DDH has not been reported to date. Given the differences in pelvic morphology between females and males, especially around the ischium [3, 11, 26, 34], if the acetabular coverage in males with DDH correlates with the rotation of the ilium and ischium, its characteristics may differ from those in females.

Therefore, using CT data from a selected population of patients with symptomatic hip dysplasia who underwent elective eccentric rotational acetabular osteotomy, we asked: (1) What are the differences in the acetabular coverage between females and males with DDH? (2) What are the differences in the rotations of the ilium and ischium between females and males with DDH? (3) What is the relationship between the rotation of the ilium and ischium and the acetabular coverage at each height in females and males with DDH?

## Patients and Methods

### Study Design and Setting

This was a retrospective, comparative study of images obtained from patients scheduled to undergo surgery at Nagoya University Hospital. This facility is a regional core hospital that accepts referred patients for eccentric rotational acetabular osteotomy aimed at treating DDH. Three hip surgeons performed eccentric rotational acetabular osteotomy, with two surgeons (YT, Y. Osawa) serving either as the lead surgeon or the first assistant. Additionally, four other hip surgeons are involved in the operations, participating on a rotating basis.

### Patients

**Fig. 1:**
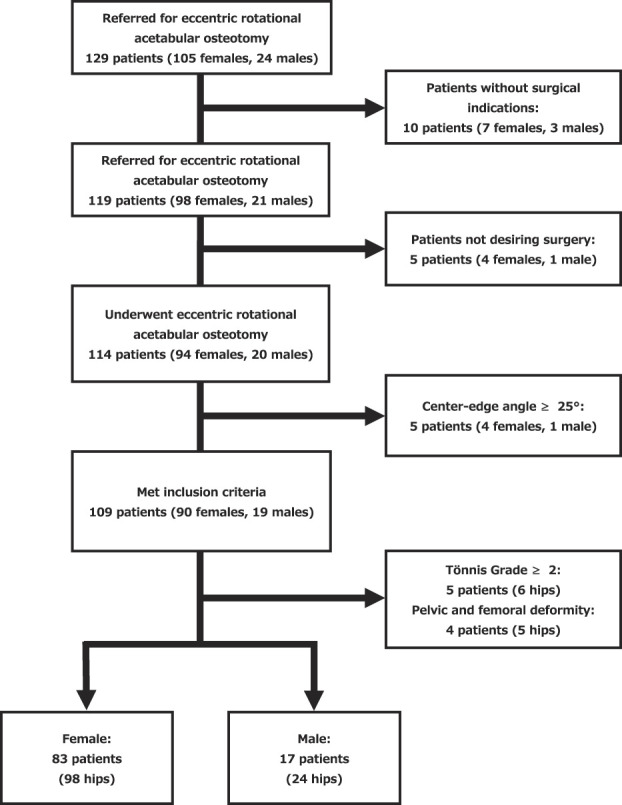
This is the patient flowchart diagram for this study.

Between January 2016 and August 2023, we evaluated 129 patients with symptomatic hip dysplasia; this group included 105 females and 24 males. Of these, we offered surgery in the form of an eccentric rotational acetabular osteotomy to 92% (119 of 129 patients [98 females and 21 males]) of patients. The remainder were treated with nonsurgical management. Of the patients offered surgery, 96% (114 of 119 patients [138 hips; 94 females, 20 males]) elected to undergo surgery. In this study, we excluded 16 hips from the patients who underwent eccentric rotational acetabular osteotomy surgery with center-edge angle ≥ 25°, Tönnis Grade 2 or higher, and significant deformities of the pelvis or femur. Ultimately, we included 100 patients (122 hips; 83 females, 17 males) in this study (Fig. 1).

### Descriptive Data

DDH was defined by a center-edge angle (CEA) of less than 25° on AP hip radiographs. The median (range) age of the patients was 40 years (10 to 58) for females and 31 years (14 to 53) for males, and the median BMI was 21 kg/m^2^ (15 to 39) for females and 24 kg/m^2^ (19 to 27) for males.

### Variables and Measurement Approaches

**Fig. 2:**
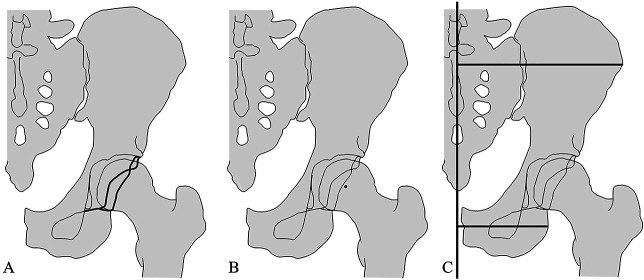
Indicators suggesting acetabular retroversion have been depicted. (**A**) The crossover sign is a finding where the anterior and posterior edges of the acetabulum intersect. (**B**) The posterior wall sign was defined as positive when the posterior wall does not extend to the center of the femoral head. (**C**) The pelvic width index is calculated as A/B.

We examined the CEA, Sharp angle, neck-shaft angle, crossover sign, posterior wall sign, and the proportion of patients with a pelvic width index [30] less than 56% on AP radiographs. Pelvic width index was defined as the ratio of the perpendicular distance from the most lateral iliac crest to the pelvis median axis to the distance from the ischium’s most lateral point to the same axis (Fig. 2). CT scans, including the pelvis and both femurs, were performed for all patients using a Toshiba Aquilion CT scanner (Toshiba Medical) with scanning parameters of 120 kV and 320 mA. The patients were supine for scanning, with images taken at 1-mm intervals from the proximal pelvis to the distal femur. All patient data were transferred to the CT-based simulation software ZedHip version 17.0.0 (Lexi Co Ltd). This software allows free-coordinate settings. The coronal plane was set so that the line connecting the teardrops on both sides was horizontal (y-axis). The axial plane was set so that the line passing through the pubic symphysis and the center of the sacrum was vertical (x-axis). For the sagittal plane, the pelvic tilt was adjusted such that both the anterior superior iliac spine and the pubic symphysis were aligned on the same line (z-axis).

**Fig. 3:**
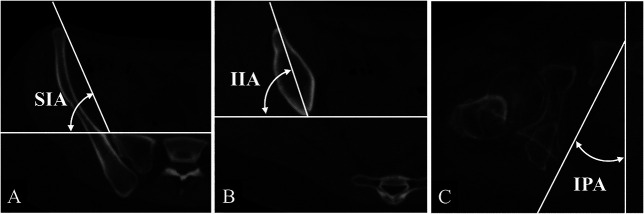
The variables related to innominate bone rotation have been depicted. (**A**) The superior iliac wing angle is determined as the point where a line connecting the inner edge of the anterior superior iliac spine and the front edge of the sacroiliac joint intersects with a horizontal line on the axial plane. (**B**) The inferior iliac wing angle is determined by the intersection of a line connecting the front part of the anterior inferior iliac spine and the rear part of the ilium with a horizontal line on the axial plane. (**C**) The ischiopubic angle is determined as an angle by the crossing of a line connecting the front upper edge of the pubic symphysis and the ischial spine, with a sagittal line on the axial plane; SIA = superior iliac wing angle; IIA = inferior iliac wing angle; IPA = ischiopubic angle.

**Fig. 4:**
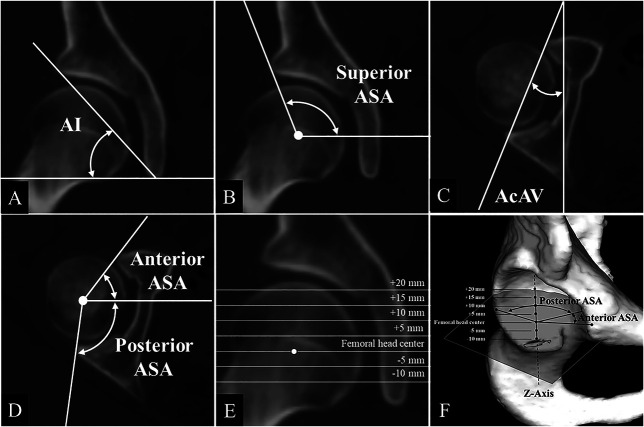
Acetabular coverage parameters have been depicted. (**A**) Acetabular inclination was measured within the coronal plane that passes through the center of the femoral head. Acetabular inclination is formed by a line connecting the upper and lower edges of the acetabulum and a horizontal line. (**B**) Superior acetabular sector angles were measured within the coronal plane that passes through the center of the femoral head. Acetabular sector angles were formed by the point where a line connecting the center of the femoral head and the edge of the acetabulum intersects with a horizontal line. (**C**) Acetabular anteversion was measured within the axial plane that passes through the center of the femoral head. It is determined as the angle created by the intersection of a line connecting the anterior and posterior edges of the acetabulum with a sagittal line. (**D**) The angles representing the anterior and posterior acetabular sector angles are determined within the axial plane that passes through the center of the femoral head. The acetabular sector angle is determined by the point where a line connecting the center of the femoral head and the edge of the acetabulum intersects with a horizontal line. (**E**) The measurements for the acetabular anteversion angle, anterior acetabular sector angles, and posterior acetabular sector angles were taken at intervals of 5 mm, starting 10 mm distal to the femoral head center and continuing up to 20 mm proximal to the femoral head center. (**F**) 3D image diagram of the posterior ASA and anterior ASA at each acetabular level; AI = acetabular inclination; ASA = acetabular sector angle; AcAV = acetabular anteversion.

The rotation of the innominate bone in the axial plane was evaluated. The rotation of the ilium was assessed using the superior iliac angle at the slice passing through the anterior superior iliac spine (Fig. 3A) and the anterior inferior iliac spine (Fig. 3B). We measured the ischiopubic angle at the slices passing through the pubic symphysis and ischial spine to observe the rotation of the ischium (Fig. 3C). Furthermore, to evaluate acetabular coverage, we measured the acetabular inclination (Fig. 4A) and the superior acetabular sector angle (Fig. 4B) in the coronal plane passing through the center of the femoral head, which indicated superior acetabular coverage. In the axial plane passing through the center of the femoral head, we measured acetabular anteversion (Fig. 4C); the anterior acetabular sector angles, which indicate the anterior coverage of the acetabulum; and the posterior acetabular sector angles, which indicate its posterior coverage (Fig. 4D). We classified the anterior and posterior acetabular coverage on slices passing through the center of the femoral head into the following categories: mild deficiency (anterior acetabular sector angles ≥ 50° and posterior acetabular sector angles ≥ 90°), anterior deficiency (anterior acetabular sector angles < 50° and posterior acetabular sector angles ≥ 90°), posterior deficiency (anterior acetabular sector angles ≥ 50° and posterior acetabular sector angles < 90°), and global deficiency (anterior acetabular sector angles < 50° and posterior acetabular sector angles < 90°) [1, 4, 15]. Furthermore, we investigated the distribution of anterior and posterior acetabular coverage. Measurements of the acetabular anteversion, anterior acetabular sector angles, and posterior acetabular sector angles were taken every 5 mm [6], starting from the slice passing through the center of the femoral head and moving distally by 10 mm, up to 20 mm proximally from the center of the femoral head (Fig. 4E, F).

### Primary and Secondary Study Outcomes

Our primary study goal was to clarify the sex differences in acetabular coverage in DDH. To achieve this, we investigated the evaluation index for acetabular retroversion in hip AP radiographs and compared the measurement for three-dimensional (3D) acetabular coverage between males and females using CT data.

Our secondary study goal was to investigate the relationship between sex-based differences in acetabular coverage in DDH and sex-based differences in pelvic morphology. To achieve this, we examined the differences in pelvic morphology between females and males and evaluated the relationship between pelvic morphology and acetabular coverage for each sex.

### Ethical Approval

This retrospective, single-center study was approved by our institution’s ethics committee.

### Statistical Analysis

The first author (HF) and the fourth author (HI) performed all measurements twice, with an interval of more than 1 month between their respective sessions. The reliabilities for both intra- and interobserver measurements for CT were good to excellent, with intraobserver reliability ranging from 0.88 to 0.93 and interobserver reliability from 0.83 to 0.94. We used the Shapiro-Wilk test to assess normal distribution, we used the Student t-test for normally distributed variables, and we used the Mann-Whitney U test for variables that were not normally distributed. We compared continuous variables related to patient demographics, innominate bone measurements, and acetabular coverage measurements between individual female and male participants. Using the Fisher exact test, we compared binary variables. Finally, we evaluated the correlations between pelvic morphology measurements (superior iliac angle, inferior iliac angle, and ischiopubic angle) and acetabular coverage (acetabular anteversion, anterior acetabular sector angles, posterior acetabular sector angles) separately for females and males using the Pearson correlation coefficient. Based on previous reports [5], we conducted an a priori power analysis, which indicated that the evaluation criterion should be the posterior acetabular sector angle with a comparison of the mean values between two different groups. With a mean difference of 6.9° ± 6.8°, we determined that we needed at least 16 patients in the female group and 16 patients in the male group to sufficiently determine differences in posterior acetabular coverage. Statistical analysis was performed using EZR version 1.41 (64‐bit) (Saitama Medical Center, Jichi Medical University), a graphical user interface for R [18], and a p value of less than 0.05 was considered statistically significant.

## Results

### Females Were More Likely to Demonstrate Anterior Deficiency, but Males Were More Likely to Demonstrate Global Deficiency

**Fig. 5:**
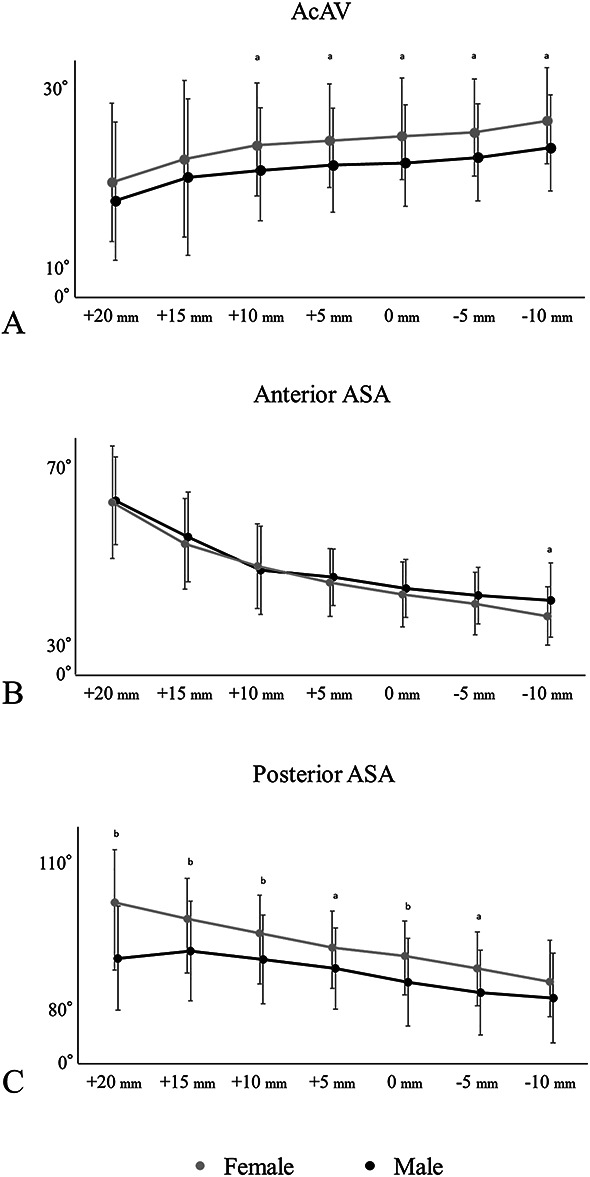
These graphs illustrate the differences between males and females in acetabular coverage at each slice of the acetabulum. (**A**) Demonstrates the disparities in acetabular anteversion between the sexes. (**B**) Demonstrates the variances in the anterior acetabular sector angle between males and females. (**C**) Demonstrates the differences in the posterior acetabular sector angle between males and females; AcAV = acetabular anteversion; ASA = acetabular sector angle. ^a^p < 0.05 and ^b^p < 0.005.

**Fig. 6:**
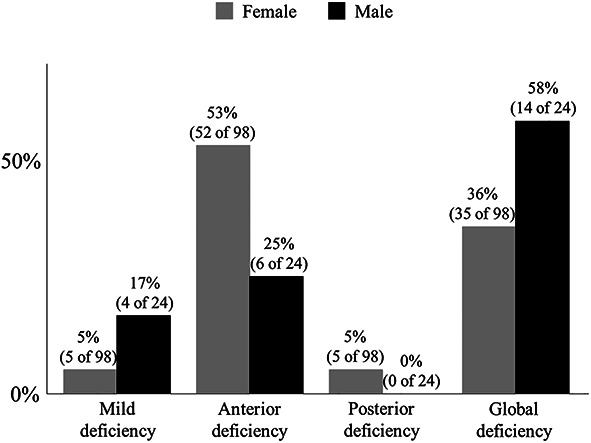
Acetabular coverage deficiency is classified as follows: mild deficiency as anterior ASA ≥ 50° and posterior ASA ≥ 90°, anterior deficiency as anterior ASA < 50° and posterior ASA ≥ 90°, posterior deficiency as anterior ASA ≥ 50° and posterior ASA < 90°, global deficiency as anterior ASA < 50° and posterior ASA < 90°; ASA = acetabular sector angle.

We observed no differences in acetabular coverage (CEA and Sharp angle) in the radiographic measurements. Suggesting acetabular retroversion, the crossover sign showed no differences between sexes, whereas the posterior wall sign (females 46% [45 of 98] versus males 75% [18 of 24], OR 3.50 [95% confidence interval (CI) 1.20 to 11.71]; p = 0.01) and pelvic width index less than 56% (females 1% [1 of 98] versus males 17% [4 of 24], OR 18.71 [95% CI 1.74 to 958.9]; p = 0.005) occurred more commonly in males than in females (Table 1). Moreover, females and males showed no differences in terms of superior acetabular sector angle (females 98° ± 10° versus males 98° ± 9°; p = 0.96) and acetabular inclination (females 49° ± 4° versus males 50° ± 9°; p = 0.31). In contrast, in the axial plane, acetabular anteversion values were smaller in males than in females for all slices (females 24° ± 5° versus male 21° ± 7°; p = 0.01 in a slice through the femoral head center), except the slices 20 mm and 15 mm proximal to the center of the head for the acetabular coverage parameter. We observed no differences for anterior acetabular sector angles except those 10 mm distal to the femoral head center for all slices. Conversely, for posterior acetabular sector angles, males showed smaller values than females for all slices (females 91° ± 7° versus males 85° ± 9°; p = 0.002 in slice through femoral head center) except for the slices 10 mm distal from the femoral head center (Fig. 5). For the distribution of the deficiency groups, females had a higher number of individuals in the anterior deficiency group (53% [52 of 98]), whereas males had a higher percentage of individuals in the global deficiency group (58% [14 of 24]) (Fig. 6).

**Table 1.** Radiographic parameters

Parameter	Females (n = 98)	Males (n = 24)	p value
Center-edge angle in °	10 ± 7	10 ± 9	0.46
Sharp angle in °	50 ± 3	48 ± 5	0.06
Neck-shaft angle in °	139 ± 14	142 ± 6	0.31
Crossover sign	4 (4)	4 (1)	> 0.99
Posterior wall sign	46 (45)	75 (18)	0.01
Pelvic width index < 56%	1 (1)	17 (4)	0.005

Data presented as the mean ± SD or % (n).

### There Were No Differences Between Males and Females Regarding Iliac Rotation, but the Ischium Was More Internally Rotated in Males

CT data indicated no differences in iliac rotation parameters (superior and inferior iliac angle) between females and males. However, the parameter evaluating the ischial rotation (ischiopubic angle) was smaller in males (females 30° ± 2° versus males 24° ± 1°; p < 0.001) (Table 2).

**Table 2.** Measurement of pelvic parameters

Parameter	Females (n = 98)	Males (n = 24)	p value
Superior iliac wing angle in °	59 ± 6	59 ± 7	0.98
Inferior iliac wing angle in °	73 ± 4	72 ± 5	0.21
Ischiopubic angle in °	30 ± 2	24 ± 1	< 0.001

Data presented as the mean ± SD.

### The Relationship Between Rotation of the Ilium and Ischium and Acetabular Coverage Differed in Females Compared With Males

**Fig. 7:**
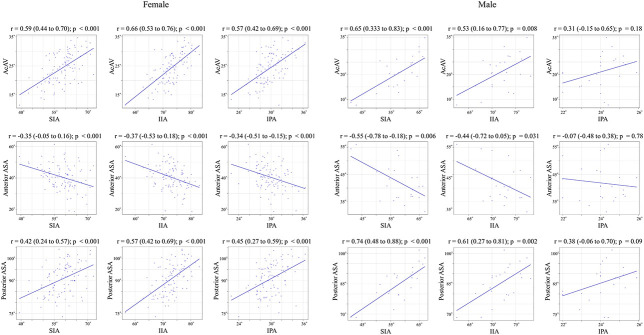
(Left) A scatter plot depicting innominate bone rotation and acetabular coverage has been created for females, showing the iliac rotation at the ASIS and AIIS levels and the ischial rotation were all found to correlate with parameters of acetabular coverage. (Right) A scatter plot depicting innominate bone rotation and acetabular coverage has been created for males, indicating iliac rotation at the ASIS and AIIS levels was found to correlate with all parameters of acetabular coverage, although no correlation was observed between ischial rotation and acetabular coverage; SIA = superior iliac wing angle; IIA = inferior iliac wing angle; IPA = ischiopubic angle; ASIS = anterior superior iliac spine; AIIS = anterior inferior iliac spine.

**Fig. 8:**
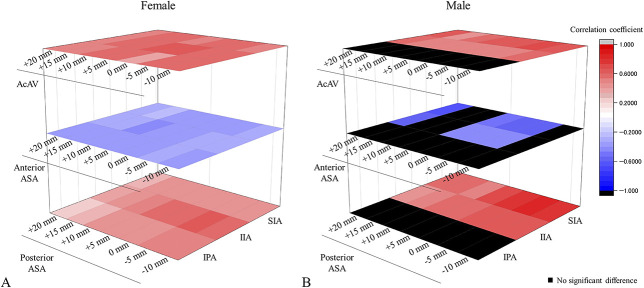
Stacked heatmaps were presented showing the correlation coefficients between 3D acetabular coverage and pelvic rotation. (**A**) The stacked heatmap of correlation coefficient between acetabular coverage and pelvic rotation in females showed the iliac rotation and ischial rotation were all found to correlate with parameters of acetabular coverage. (**B**) The stacked heatmap of correlation coefficient between acetabular coverage and pelvic rotation in males demonstrated iliac rotation was found to correlate with many parameters of acetabular coverage, although no correlation was observed between ischial rotation and acetabular coverage; ASA = acetabular sector angle; SIA = superior iliac angle; IIA = inferior iliac angle; IPA = ischiopubic angle. A color image accompanies the online version of this article.

In females, we observed a correlation between innominate bone rotation and acetabular coverage as follows: superior iliac angle versus acetabular anteversion (r = 0.59 [95% CI 0.44 to 0.70]) versus anterior acetabular sector angle (r = -0.35 [95% CI -0.05 to 0.16]) and versus posterior acetabular sector angle (r = 0.42 [95% CI 0.24 to 0.57]); inferior iliac angle versus acetabular anteversion (r = 0.66 [95% CI 0.53 to 0.76]) versus anterior acetabular sector angle (r = -0.37 [95% CI -0.53 to 0.18]) and versus posterior acetabular sector angle (r = 0.57 [95% CI 0.42 to 0.69]); ischiopubic angle versus acetabular anteversion (r = 0.57 [95% CI 0.42 to 0.69]) versus anterior acetabular sector angle (r = -0.34 [95% CI -0.51 to -0.15]) and versus posterior acetabular sector angle (r = 0.45 [95% CI 0.27 to 0.59]) (all p < 0.001) (Table 3). Thus, we found that in females, the external rotation of the ilium (indicated by a decrease in the superior iliac angle and inferior iliac angle) and the internal rotation of the ischium (indicated by a decrease in the ischiopubic angle) were associated with an increase in the anterior acetabular sector angle and a decrease in the posterior acetabular sector angle. In contrast, acetabular coverage in males showed a correlation with the iliac angle as follows: superior iliac angle versus acetabular anteversion (r = 0.65 [95% CI 0.33 to 0.83]) versus anterior acetabular sector angle (r = -0.55 [95% CI -0.78 to -0.18]) and versus posterior acetabular sector angle (r = 0.74 [95% CI 0.48 to 0.88]); inferior iliac angle versus acetabular anteversion (r = 0.53 [95% CI 0.16 to 0.77]) versus anterior acetabular sector angle (r = -0.44 [95% CI -0.72 to 0.05]) and versus posterior acetabular sector angle (r = 0.61 [95% CI 0.27 to 0.81]); all p < 0.05), and no correlation was observed with ischiopubic angle (Table 3). Thus, we found that in males, external rotation of the ilium, indicated by a decrease in the superior iliac angle and inferior iliac angle, was associated with an increase in the anterior acetabular sector angle and a decrease in the posterior acetabular sector angle, whereas changes in the ischiopubic angle were not associated with acetabular coverage (Fig. 7). We observed this trend across all levels of the acetabulum (Fig. 8).

**Table 3.** Correlation between subgroups at the femoral head center slice

	Females (n = 98)		Males (n = 24)		
	Correlation coefficient (95% CI)	p value	Correlation coefficient (95% CI)	p value
Correlations with SIA				
AcAV in °	0.59 (0.44 to 0.70)	< 0.001	0.65 (0.33 to 0.83)	< 0.001
Anterior ASA in °	-0.35 (-0.05 to 0.16)	< 0.001	-0.55 (-0.78 to -0.18)	0.006
Posterior ASA in °	0.42 (0.24 to 0.57)	< 0.001	0.74 (0.48 to 0.88)	< 0.001
Correlations with IIA				
AcAV in °	0.66 (0.53 to 0.76)	< 0.001	0.53 (0.16 to 0.77)	0.008
Anterior ASA in °	-0.37 (-0.53 to 0.18)	< 0.001	-0.44 (-0.72 to 0.05)	0.03
Posterior ASA in °	0.57 (0.42 to 0.69)	< 0.001	0.61 (0.27 to 0.81)	0.002
Correlations with IPA				
AcAV in °	0.57 (0.42 to 0.69)	< 0.001	0.31 (-0.15 to 0.65)	0.18
Anterior ASA in °	-0.34 (-0.51 to -0.15)	< 0.001	-0.07 (-0.48 to 0.38)	0.78
Posterior ASA in °	0.45 (0.27 to 0.59)	< 0.001	0.38 (-0.06 to 0.70)	0.09

AcAV = acetabular anteversion angle; ASA = acetabular sector angle; SIA = superior iliac wing angle; IIA = inferior iliac wing angle; IPA = ischiopubic angle.

## Discussion

When performing eccentric rotational acetabular osteotomy for DDH, it is necessary not only to optimize superior acetabular coverage but also to optimize anterior and posterior acetabular coverage. It is known that in females with DDH, the rotation of the acetabulum in the axial plane is associated with acetabular coverage. However, the relationship between acetabular coverage and pelvic morphology in males with DDH remains unclear. We aimed to clarify the sex-based differences and relationship in pelvic morphology and acetabular coverage by comparing the CT data of females and males with DDH before eccentric rotational acetabular osteotomy. The findings revealed a higher prevalence of the posterior wall sign and a greater percentage of males with a pelvic width index less than 56% compared with females, which suggests acetabular retroversion is more common in males with DDH. Although no sex-related differences were observed in the anterior acetabular coverage at each acetabular height, in terms of posterior coverage, males with DDH had lower acetabular coverage than females with DDH.

Additionally, there were more males with DDH than females with DDH in the global deficiency group. In our analysis of the correlation between acetabular coverage and innominate rotation, acetabular coverage was associated with the ilium and ischium rotation in females with DDH. However, in males with DDH, acetabular coverage was associated with ilium rotation but was not correlated with ischium rotation. Our thinking that differences in pelvic morphology between sexes are related to the sex-based differences in acetabular coverage was correct. When performing eccentric rotational acetabular osteotomy for males with DDH, it might be essential to adjust the rotation of the bone fragments to ensure adequate posterior acetabular coverage is maintained.

### Limitations

This study has several limitations. First, the number of males with DDH was small. This study used sample size calculations to determine the mean posterior acetabular coverage difference between females and males, based on a previous study [5]. However, a larger sample size is necessary to determine the correlation coefficient accurately. Should the number of males with DDH increase in the future, a reevaluation will be required. Nevertheless, to the best of our knowledge, this study had the largest number of cases available to compare DDH morphology between females and males. Second, the pelvises of females and males exhibited a distinct dimorphic pattern. Although the methodologies employed in this study align with the established criteria from previous publications evaluating pelvic morphology, the assessment point for the pelvic morphology used in this study may differ for each sex. Nevertheless, contemporary osteotomies and THA use identical coordinate configurations, irrespective of sex. Thus, the insights gained from this study hold significance for orthopaedic clinical practice [32]. Third, the scope of this study was exclusively confined to patients classified as Asian, and hip morphology may vary across racial demarcations [2].

Nonetheless, within identical racial cohorts, the propensity for morphological distinctions attributable to sex is consistent [2]. Fourth, this study involved DDH cases that are suitable for eccentric rotational acetabular osteotomy and classified as Crowe Type I. How this approach is applicable to cases such as borderline dysplasia, asymptomatic DDH, or high dislocation of the hip remains unclear. It has been reported that the correlation between acetabular coverage and pelvic morphology also applies to groups of hips without symptoms in the normal population [4]. However, further examination is necessary in patients with high hip dislocation. Fifth, the clinical utility of classifying acetabular deficiency based on minor differences in coverage remains unclear. Our categorization of acetabular deficiency divides the continuous variable of acetabular coverage and may be distinguishing between groups based on very small, almost negligible differences. Furthermore, the clinical significance of this categorization is not clear. Ibrahim et al. [12] showed that postoperative deficiency in posterior coverage was associated with activities of daily living. This implies that cases with a preoperative deficiency in posterior acetabular coverage might require more restoration of posterior coverage. Therefore, this classification could potentially become a clinical indicator in the future. Sixth, the importance of this study may vary with the osteotomy type performed. The acetabular osteotomy method can influence the ease with which bone fragments can be rotated anteriorly and posteriorly. As a result, leveraging the insights from this study to adequately cover the anterior and posterior acetabulum may necessitate an evaluation of how well these findings align with the techniques employed at individual institutions. Finally, the results differ only at the most distal part of the acetabulum from the other slices. In this study, the acetabulum was sliced in the axial plane every 5 mm for evaluation. Similar results were obtained in almost all slices, but the evaluation at the most-distal axial plane was different from the other results. This may be due to many cases where the acetabular fossa enters from the front at the most distal height, and some are outside the joint. After eccentric rotational acetabular osteotomy, because the distal acetabular coverage rises toward the proximal side of the joint, more careful studies on the distal acetabular coverage may be necessary.

### Females Were More Likely to Demonstrate Anterior Deficiency, but Males Were More Likely to Demonstrate Global Deficiency

Our study showed a 3D assessment of the pelvic bony morphology in symptomatic hips in males with DDH. Our results indicated that, in nearly all axial slices, except for the most distal axial slice, the posterior wall of the acetabulum in males with DDH was lower than that in females with DDH. However, no significant differences in anterior coverage were noted between the sexes. The acetabular version was also lower in males than in females with DDH. Thus, the reduced acetabular version observed in males with DDH compared with that in females with DDH may be attributed to their lower posterior coverage. These findings suggest that acetabular coverage features in males with DDH may indicate a potential risk for acetabular retroversion. Although the anterior acetabular coverage remains the same, decreased posterior coverage increases the hip instability risk and may lead to labral tears. Furthermore, reducing posterior coverage in males with DDH can also be helpful when rotating the acetabular fragment during eccentric rotational acetabular osteotomy. A retrospective study suggested that deficient posterior acetabular coverage after osteotomy was associated with reduced activities of daily living [12]. During eccentric rotational acetabular osteotomy, the acetabular fragment is sometimes rotated anteriorly to compensate for insufficient anterior acetabular coverage. Furthermore, in males with DDH, it may be necessary to rotate the acetabular fragment in such a way that it also restores coverage to the posterior aspect of the acetabulum. However, suppose the posterior coverage deficiency in males with DDH is due to the shallowness of the acetabulum socket, leading to a reduced area of acetabular cartilage. In that case, precise bone fragment rotation might be necessary to cover the acetabulum’s anterior and posterior coverage adequately. The differences in the depth of acetabular coverage and the area of acetabular cartilage between sexes in DDH are not apparent [33]. However, precise movement of the bone fragment using navigation might be beneficial when performing eccentric rotational acetabular osteotomy on males with DDH [15]. Building on these insights, a few reports have examined the anterior and posterior acetabular coverage in detail [5]. Partial acetabulum evaluations showed that both the posterior [5] and posterosuperior [24] acetabular coverage in males with DDH were lower than those in their female counterparts. However, studies reporting these findings included fewer than 10 hips each. This study calculated the sample size based on previous studies [5] and confirmed that 24 males constituted a sufficient sample size. In the future, it will be necessary to evaluate the acetabular coverage of both females and males in various limb positions, such as standing, supine, and walking. In cases of asymptomatic hips, females tend to tilt anteriorly during walking compared with males [20]. It is necessary to investigate whether this characteristic also applies to patients with DDH and to evaluate the impact of various limb positions for acetabular coverage.

### There Were no Differences Between Males And Females Regarding Iliac Rotation, but the Ischium Was More Internally Rotated in Males

In our study, although no differences were observed in ilium rotation (superior iliac angle and inferior iliac angle) between females and males with DDH, the ischium rotation (ischiopubic angle) in males with DDH was predominantly rotated internally compared with females with DDH. These results coincided with the characteristics of sex-based differences in the pelvic morphology of asymptomatic hips [11]. The pelvic morphology of females and males with asymptomatic hips exhibits distinct dimorphism [33]. Although differences in pelvic morphology are present from birth, the pelvic morphology of females with asymptomatic hips, compared with that of males with asymptomatic hips, shows the prominent external rotation of the ischium between 15 and 25 years of age [11]. This period coincides with when the estradiol concentration in females increases [26], indicating changes in preparation for childbirth. Rotation of the ischium has been shown to correlate with acetabular coverage [4], and the differences in ischial rotation between males and females with DDH may be related to sex-based differences in acetabular coverage.

### The Relationship Between Rotation of the Ilium and Ischium and Acetabular Coverage Differed in Females Compared With Males

In females, the iliac and ischial rotation was correlated with proximal-to-distal acetabular coverage. In contrast, in males, acetabular coverage correlated with the iliac rotation across all slices, with no correlation observed with the ischium. When performing eccentric rotational acetabular osteotomy, rotating the acetabular bone fragment laterally brings the acetabulum’s distal area closer to the acetabulum’s center. Therefore, it is essential to understand the acetabular coverage in 3D. Like females with asymptomatic hips, innominate bone rotation in females with DDH correlates with acetabular coverage [4]. At first, we speculated that the proximal slice of the acetabulum would be correlated with the iliac rotation, whereas the distal slice would be correlated with ischial rotation. Additionally, we thought that the posterior coverage of the acetabulum has been speculated to increase as the ischium rotates externally during puberty. However, this characteristic did not become more pronounced distally in the acetabulum but correlated with the acetabulum as a whole. Compared with females, males do not have significant morphological changes in the pelvis during puberty [11]. From a clinical perspective, even if the pelvic bone fragment is laterally rotated to a similar extent in both females and males during eccentric rotational acetabular osteotomy, males may still have less posterior coverage compared with females. In simpler terms, even when males and females with DDH have similar degrees of iliac rotation, the greater external rotation of the ischium in females may result in increased posterior acetabular coverage in women (Supplemental Fig. 1; http://links.lww.com/CORR/B305) compared with less posterior coverage in males (Supplemental Fig. 2; http://links.lww.com/CORR/B305). This is because the external rotation of the ischium in females is associated with increased posterior acetabular coverage in females. In other words, it is essential to take note of this when performing eccentric rotational acetabular osteotomy. When performing eccentric rotational acetabular osteotomy for males with DDH, properly adjusting the rotation of acetabular bone fragments might prove advantageous in addressing insufficient posterior acetabular coverage.

### Conclusion

In summary, we investigated sex-based differences in the relationship between innominate bone rotation and acetabular coverage in patients with DDH before eccentric rotational acetabular osteotomy. Males more frequently had acetabular retroversion compared with females because of the smaller posterior acetabular coverage in males. Females exhibited greater external rotation of the ischium compared with males, and this external rotation was correlated with increased posterior acetabular coverage. When performing eccentric rotational acetabular osteotomy for males with DDH, it might be advisable to carefully rotate the bone fragments to avoid insufficient posterior acetabular coverage. Future studies might investigate the differences in acetabular coverage between the sexes when assessed in various limb positions to rotate the bone fragment appropriately.
